# Cutaneous Carcinosarcoma with Metastasis to the Parotid Gland

**DOI:** 10.1155/2014/173235

**Published:** 2014-09-25

**Authors:** Tze Ling Loh, Jeanne Tomlinson, Ronald Chin, Guy D. Eslick

**Affiliations:** ^1^Department of Surgery, St Vincent's Hospital, 390 Victoria Street, Darlinghurst, NSW 2010, Australia; ^2^Department of Anatomical Pathology, Westmead Hospital, Cnr Hawkesbury Road & Darcy Road, Westmead, NSW 2145, Australia; ^3^Department of Otolaryngology, Head and Neck Surgery, Nepean Hospital, Derby Street, Penrith, NSW 2750, Australia; ^4^Department of Surgery, The Whiteley-Martin Research Centre, Nepean Hospital, Derby Street, Penrith, NSW 2750, Australia

## Abstract

Cutaneous carcinosarcoma is a rare malignancy that exhibits both mesenchymal and epithelial components. It is similar to nonmelanoma skin cancers in terms of risk and prognostic factors. However, these malignancies are known to have a propensity for local recurrence and metastasis, even with adequate resection margins. Here we report a case of metastatic cutaneous carcinosarcoma to the parotid gland and review the relevant literature.

## 1. Introduction

Carcinosarcomas are rare malignancies that express both mesenchymal and epithelial components. They have been described in various parts of the body, including the bladder, ovaries, breast, uterus, lung, and larynx. Cutaneous forms of carcinosarcoma are particularly rare. Here, we present a case of cutaneous carcinosarcoma and review the relevant literature. Our case is the first reported metastatic carcinosarcoma to the parotid gland—this case demonstrates clearly the great propensity of this tumour for early distant metastasis.

## 2. Case Presentation

**Figure 1:**
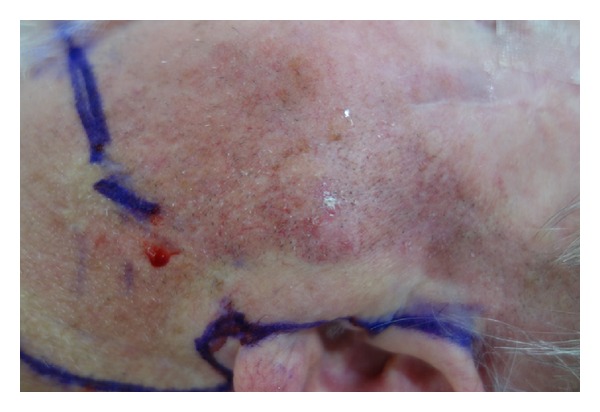
Pre-op photo showing left parotid lesion.

A 75-year-old man was referred to our head and neck oncology service with a 2 × 2 cm left-sided preauricular lump (Figure 1). The lump was firm to palpation. There was no fixation to the overlying skin or underlying tissue. There was no palpable cervical lymphadenopathy. There were no associated cranial nerve palsies.

**Figure 2:**
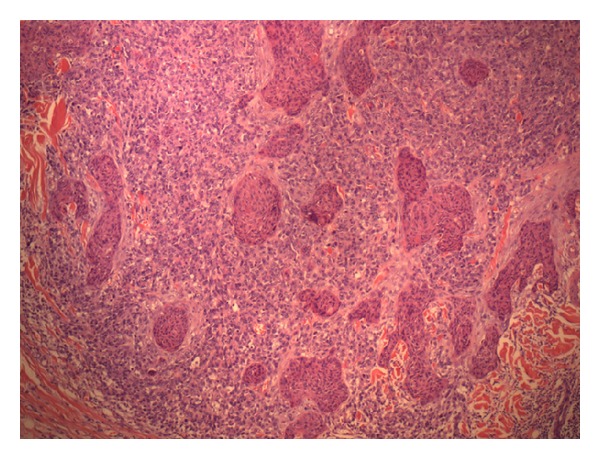
Squamous islands and undifferentiated cells H&E ×40.

**Figure 3:**
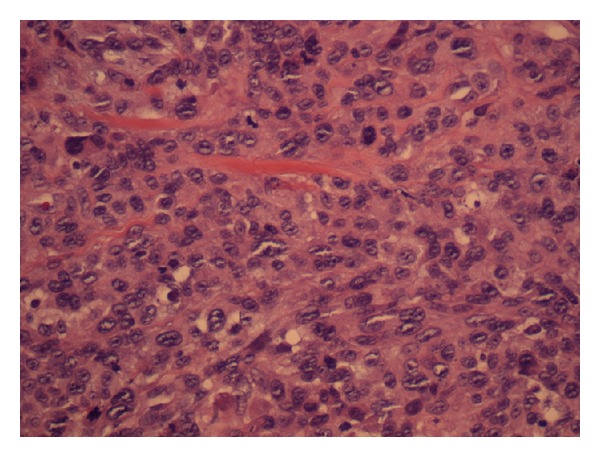
Undifferentiated cells H&E ×200.

**Figure 4:**
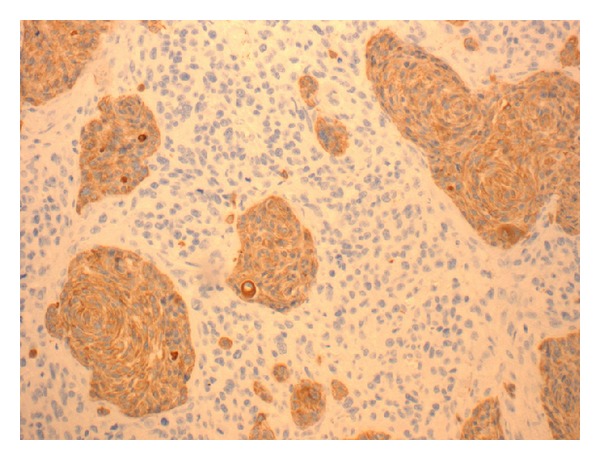
Islands of squamous cells staining positive for cytokeratin. Cytokeratin AE1/3 ×100.

The patient had a skin lesion excised from his left temporal region 6 months prior to this presentation. This initial skin lesion was described as “granuloma-like” and grew quickly over a period of 2 months to a size of 1.7 cm. The histopathology of the left temporal lesion showed a carcinosarcoma. It contained islands of malignant squamous epithelium surrounded by sheets of large undifferentiated cells (Figures 2 and 3). Cytokeratin AE1/3 marked the squamous epithelial islands and occasional undifferentiated cells (Figure 4). The tumour was confined to the deep reticular dermis with a measured depth from the epidermal granular layer of 3.5 mm. No vascular or perineural invasion was seen. The tumour was excised with a margin of at least 1 mm. The patient subsequently underwent superficial radiotherapy to the tumour bed at a dose of 51 Gy in 17 fractions.

The patient had a background history of type 2 diabetes, hypertension, and a cardiac pacemaker. He was a nonsmoker and did not have a significant alcohol history. There was no significant family history of cancers.

## 3. Investigations

Staging investigations, including a PET-CT study and a diagnostic contrast CT study of the head and neck, were performed.

**Figure 5:**
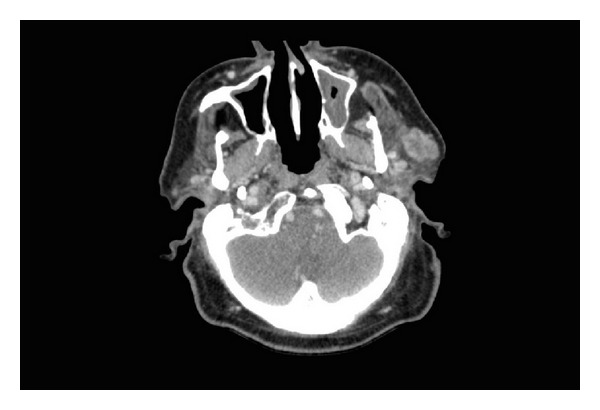
Preoperative CT showing parotid gland lesion.

CT of the neck showed an irregularly heterogeneously enhancing 2.4 × 1.7 cm lesion arising from the left parotid gland (Figure 5). There was no evidence of bony involvement. There were no lesions in the lung parenchyma.

There was intense FDG uptake corresponding to the left parotid gland lesion. There was also low-grade uptake in the left level 2A lymph nodes. There was otherwise no other distant FDG-avid uptake in other parts of the body.

A fine needle aspiration biopsy of the parotid lesion showed large sheets of highly pleomorphic epithelial cells, with an appearance suggestive of an epithelial malignancy.

## 4. Treatment

The patient underwent a left parotidectomy and a left modified radical neck dissection of levels II, III, and IV, with facial nerve monitoring after discussion of his case at a multidisciplinary head and neck oncology meeting. The left facial nerve and its branches were identified and preserved with blunt dissection. The parotid gland containing the tumour was resected. The left spinal accessory nerve was preserved. He subsequently underwent adjuvant radiotherapy treatment.

## 5. Outcome

The patient had temporary left-sided frontalis muscle dysfunction postoperatively. His postoperative recovery was otherwise unremarkable.

Histopathology specimens included the left parotid gland containing a 30 × 23 × 15 mm firm, pale nodular tumour as well as 3 periparotid lymph nodes. A further 37 lymph nodes were recovered from the left levels 2 and 3 dissections.

**Figure 6:**
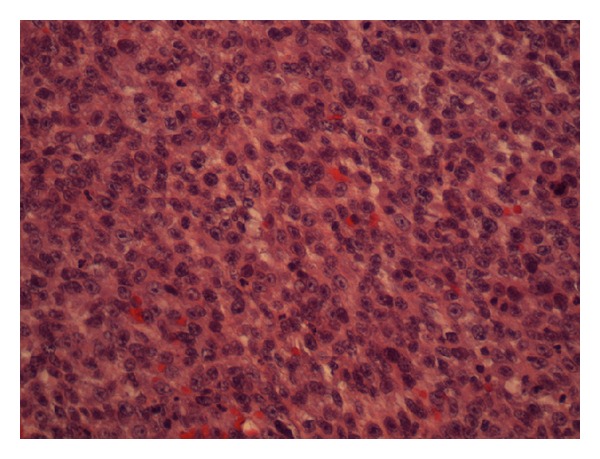
Undifferentiated sarcomatous cells in parotid specimen. H&E ×200.

Sections of the tumour showed sheets of undifferentiated, highly pleomorphic plump epithelioid and spindled cells with appearances similar to the undifferentiated sarcomatous component in the skin excision (Figure 6). Very occasional cells stained for cytokeratin AE1/3, as seen previously. Islands of squamous cells were not seen. The tumour was not surrounded by lymphoid tissue. All lymph nodes examined showed no evidence of metastatic tumour.

## 6. Follow-Up

**Figure 7:**
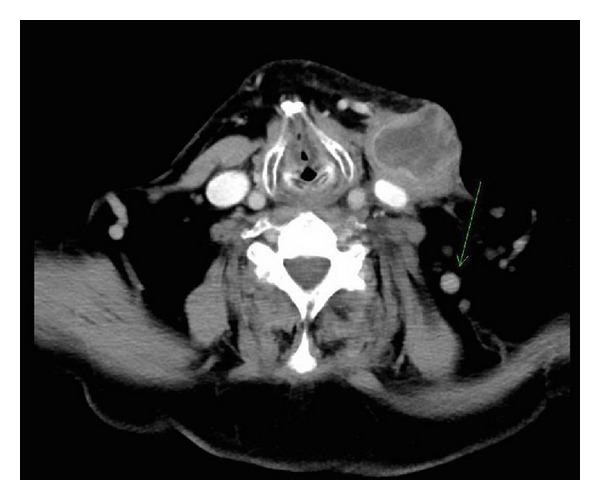
CT scan 6 months post-op, showing haemorrhagic node and level V lymphadenopathy (arrow).

The patient was referred back to our clinic 6 months after the parotid surgery with a left-sided neck lump. His CT scan was consistent with a haemorrhagic lymph node and enlarged nodes in the left neck (Figure 7). A repeat staging PET-CT was performed, which showed areas of increased uptake in the node and multiple FDG-avid nodules in the lungs, consistent with metastatic disease. The patient was referred to medical oncology for chemotherapy.

Carcinosarcoma of the skin is a rare malignancy, with about 50+ cases reported in the literature. Our review of the literature revealed no clear consensus on the criteria for histopathological diagnosis of carcinosarcoma. The disease has also been referred to as spindle cell carcinoma [1], sarcomatoid carcinoma [2–5], metaplastic carcinoma [6], and pseudosarcoma [7, 8]—adding further confusion to diagnosis. The variance in nomenclature could be attributed to conflicting views in the pathogenesis of carcinosarcoma.

## 7. Discussion

Four main theories of pathogenesis have been proposed based largely on the pathology seen in carcinosarcoma of other organs such as the female genital tract and lungs [9–12]. The first, the* collision tumour theory*, suggests that two independent tumours have collided. This is based on the observation that skin cancers and superficial malignant fibrous histiocytomas are commonly seen in patients with sun-damaged skin. The second, the* composition theory*, suggests that the mesenchymal component represents a pseudosarcomatous reaction to the epithelial malignancy. The third, the* combination theory*, holds that both malignant components arise from a common pluripotential stem cell that undergoes divergent differentiation. The fourth, the* conversion/divergence theory*, argues that the sarcomatous component represents a metaplastic sarcomatous transformation of the epithelial component. Immunohistochemical, ultrastructural, and molecular genetic studies suggest and favour the notion of monoclonality in carcinosarcoma. Identical* p53 *and* k-RAS *mutations have been identified in both epithelial and mesenchymal components, suggesting an alteration early in the histogenesis with late transformation or degeneration of the epithelial component into the sarcomatous component.

In a meta-analysis in 2005, Tran et al. selected cases that demonstrated the following: (1) concurrent presence of malignant epithelial and sarcomatoid components, (2) no keratin expression in the mesenchymal components, and (3) no transition zones from carcinoma to sarcoma [13]. Adnexal carcinosarcoma tended to present in younger patients with a history of a long-standing skin lesion, whereas the epidermal subtype was associated with elderly patients with a history of sun exposure. The study suggested that adnexal variants have poorer outcomes, possibly related to the more advanced stage of disease at diagnosis. However, on multivariate analysis, it was shown that the 5-year survival was only significantly related to tumour size, duration, patient age, and lymph node involvement. This clinical profile is similar to nonmelanoma skin cancer in many aspects. They tend to occur in elderly patients, especially in the head and neck areas that are exposed to UV radiation from sunlight.

Cutaneous carcinosarcoma has been reported to metastasise to many sites, including the tongue [14], brain, lungs, kidney, adrenal glands, bones, muscles, and lymph nodes. To the best of our knowledge, this is the first case of metastatic cutaneous carcinosarcoma to the parotid gland. This is not a surprising site for metastasis as temporal skin lesions commonly metastasise to intra- and periparotid nodes. In this case, the propensity for early metastasis is highlighted by the fact that our patient had metastatic disease despite adequate resection margins and adjuvant radiotherapy. The metastatic component is usually carcinomatous, but sarcomatous metastasis has been reported in some cases [1, 15, 16]. The impact of this on prognosis is unclear.

Interestingly, there is a high rate of local recurrence in these malignancies in the literature. The high rate of recurrence in the literature is despite short follow-up periods in the literature. This possibly suggests that the long-term recurrence rate could be higher than reported. Interestingly, in the case by Quay et al., the malignancy was found in the lung after a disease-free interval of 5 years [17, 18]. Based on this, we would recommend that these patients have prolonged regular follow-up. The metastatic lesion in our patient's parotid demonstrated intense FDG uptake on PET-CT. In patients with high risk of recurrence, PET-CT may be a means of surveillance for distant metastasis after resection or for prognostication purposes.

## 8. Conclusion

Cutaneous carcinosarcoma is a rare malignancy with a clinicopathological profile similar to nonmelanoma skin cancer. Careful histopathological evaluation is the key to diagnosing this disease. It is an aggressive tumour with a high propensity to recur locally and metastasise despite adequate resection margins. Complete surgical excision, with regular follow-up, is essential in the long-term management. The benefit of adjuvant chemotherapy or radiotherapy is uncertain, given the paucity of literature in this area.
